# When Should Commence Dialysis: Focusing on the Predialysis Condition

**DOI:** 10.5812/numonthly.5435

**Published:** 2013-03-30

**Authors:** Stefano Maffei, Silvana Savoldi, Giorgio Triolo

**Affiliations:** 1 SCDO Nephrology and Dialysis, C.T.O./Maria Adelaide Hospital, Turin, Italy; 2 Nephrology and Dialysis Unit, Cirié Hospital, Turin, Italy

**Keywords:** Glomerular Filtration Rate, Kidney Failure, Chronic, Renal Dialysis

## Abstract

The prevalence of chronic kidney disease (CKD), as defined by the NFK-KDOQI (the national kidney foundation kidney disease outcomes quality initiative) guidelines, is a glomerular filtration rate less than 60 mL/min/1.73 m^2^ or the presence of microalbuminuria. CKD is increasing worldwide, leading to an increased risk of cardiovascular disease. There is general agreement on the importance of an early referral to a nephrologist and predialysis educational programs. Establishing the protocol for an early approach may assist in preventing the progression, and the most common complications of renal disease.

Predialysis education helps patients in order to choose a renal replacement therapy (hemodialysis, peritoneal dialysis, transplantation) and improve their quality of life. Furthermore, adequate predialysis care allows the nephrologist to promptly prepare for vascular or peritoneal treatment. Regrettably, patients are often referred to the nephrologist when renal failure is already fall in the advanced stage. This is caused primarily by non-nephrologists failing to identify patients at risk for imminent renal failure. Furthermore, they may be defining the patient’s degree of renal failure according to the KDOQI classification. To further complicate matters, the serum creatinine alone does not provide an adequate estimate of renal function; however, both the MDRD (the modification of diet in renal disease) equation and the Cockcroft-Gault formula permit the more reliable and accurate estimation of the all-important glomerular filtration rate (GFR). Using the MDRD equation, the KDOQI guidelines recommend referral when GFR is less than 30 mL/min/1.73 m^2^. Late nephrology referral is an independent risk factor for early death while on dialysis; it is also associated with a more frequent use of temporary catheters, particularly in the elderly individuals. This subject underlines the importance of a multidisciplinary predialysis approach that may bring additional benefits – beyond referral to a nephrologist – including a reduced hospitalization period and a lower mortality rate.

The KDOQI guidelines recommend evaluating the benefits and risks of starting renal replacement therapy when patients reach stage 5 (estimated GFR less than 15 mL/min/1.73 m^2^), although the ideal period for initiation of the replacement therapy remained a source of debate.

## 1. Background

The incidence of chronic renal failure is increasing worldwide (1). K/DOQI clinical practice guidelines for chronic kidney disease defines the presence of CKD when the GFR is less than 60 mL/min/1.73 m^2^ or there is microalbuminuria (2). At this level of renal function, patients develop an increased risk of complications such as secondary hyperparathyroidism, anemia and hypertension (2). Recent studies suggest that patients with chronic kidney disease may suffer an increased risk of death and hospitalization for cardiovascular diseases (3).

## 2. The Role of the “Early Referral”

It is common opinion that early referral to a nephrologist and proper patient education are essential and complementary. The early referral of an outpatient with CKD at risk of end-stage renal disease to a nephrologist makes it possible to implement all procedures and treatments to control the progression of the disease. Such procedures include tight control of blood pressure, censoring the use of drugs that inhibit the renin-angiotensin system, the importance of lifestyle modification such as the necessity of exercise, cessation of smoking, a low-protein diet intake, and the use of statins and optimal glycemic control in patients who suffer diabetes. An early refer to the nephrologist can also prevent complications of chronic kidney disease such as malnutrition, anemia, osteodystrophy and acidosis that occur various stages of renal failure (2, 4). It is also easier to mitigate comorbidities such as cardiovascular diseases and neuropathies.

The follow-up patients with chronic renal failure it is necessary to allow patient to be prepared for dialysis through an educational program, which allows the choice of treatment modalities. The beginning of hemodialysis treatment must be scheduled within the required time and while the vascular access already established.

Patient education is crucial because their awareness of the disease and treatment modalities is likely to engender greater collaboration between patient and medical staff in terms of compliance and timely reporting of complications. Patient enrollment in an outpatient program also seems to improve the educational rehabilitation and quality of life (5).

## 3. The Problem of the "Late Referral"

Ideally the patient with chronic renal failure is identified early in the process; unfortunately, more often than not, the nephropathic patient comes to the attention of the nephrologist in advanced stages of the disease or when a replacement treatment is required. A recent study (6) revealed that the creatinine measurements is prescribed only in 20% of patients who are at risk (elderly, hypertensive, diabetic); the low frequency which is required for the evaluation of renal function reflects the inadequate attention of the general practitioner about kidney disease and consequently the difficulty in identifying early-stage patients with renal insufficiency.

In addition to the early recognition of the nephropathic subjects, it is essential that the terminology of renal failure is standardized and disease has been classified according to the stages K/DOQI (1). It is therefore necessary to know the creatinine, age, gender, race and body weight in order to calculate the glomerular filtration rate since the creatinine value alone should not be used to determine the level of renal function. The formulas Cockcroft-Gault and MDRD seem to respond better these requirements (4).

According to US guidelines, patients who are at stage IV (GFR < 30 mL/min/1.73 m^2^), should be directed to a nephrology clinic’s where they can be informed about their disease and proper treatment (hemodialysis, peritoneal dialysis, transplant) (4). The delay in directing the patient to the nephrologist is an independent risk factor for early death on dialysis (7, 8).

Elderly patients more frequently arrive late to the nephrologist and likewise initiate dialysis with a temporary access; commencing dialysis with a temporary catheter which increases the risk of death (8). Unfortunately, some of the patients treated at the outpatient clinics start dialysis in the emergency department due to inappropriate planning of dialysis or of creating vascular access (9). Therefore it is very important to give the patients access to a clinic dedicated to pre-dialysis where nurses and doctors, as well as dietitians, psychologists, etc., are able to provide the necessary education and collaborate in planning the start time of dialysis. A patient who is treated in pre-dialysis in a multidisciplinary clinic may suffer fewer hospitalization period as well as better chance to start dialysis with a permanent vascular access in addition to a significantly higher survival rate, as compared to patients who are in follow-up in an outpatient standard clinic (10, 11). In our clinics, patients are more often informed about hemodialysis compared to peritoneal dialysis or transplantation, and this affects the subsequent treatment modalities penalizing the choice of peritoneal dialysis (12).

Another important point is that the patient with chronic renal failure should be promptly dispatched to the nephrology center which will be responsible for their dialysis treatment. This will allow for the maintenance of as much continuity of treatment as possible in different phases of the disease, particularly in the start-up period up to the dialysis.

The method of vascular access affects morbidity and mortality in hemodialysis. The failure of the fistula can be caused by the presence of cardiovascular disease, the use of temporary catheters, the late referral of the patient and the early puncture of the fistula are the main factors (13). For these reasons, it is recommended that at least a month pass for the maturation of the fistula prior to the first injection is made.

## 4. Timing of Dialysis Initiation

According to the K/DOQI guidelines, when the patient reaches the stage V of renal failure (GFR < 15 mL/min/1.73/m^2^), the risks and benefits of dialysis treatment should be evaluated (4). Theoretical considerations support initiating dialysis with a GFR of approximately 10 mL/min/1.73 m^2^. In 2003, the US average GFR at the start of dialysis was 9.8 mL/min/1.73 m^2^ (USRDS). This value reflects lower average values for young adults and higher values for children and the elderly (14). In a study, 85% of patients innitiated treatment with a GFR less than 10 mL/min/1.73m^2^ and about 20% with a GFR less than 5 mL/min/1.73 m^2^ (15).

Actully, it is difficult to rationalize starting dialysis only on the basis of specific levels of GFR and this issue has recently been debated as evidenced in the international guidelines (Table 1) and in the observational studies or cases-controls. Some of these studies and cases supported an early initiation of dialysis in order to improve the survival rate of the patient, the quality of life and to diminish the uraemia complications (16, 17), while others suggest that an early initiation is not associated with clear survival advantages and could actually be deleterious to patient’s health (18-24). Patients who starting dialysis at higher levels of GFR in fact seem to have an increased risk of death not fully explained by the concomitant presence of associated pathologies (25).

**Table 1.** Levels of GFR at Which Is Recommended Dialysis to Start: International Guidelines

Guidelines	GFR Starting Dialysis	Comments
**USA: K/DOQI (2006) **		
	< 15 mL/min/1.73 m^2^	When patients reach stage 5 CKD, nephrologists should evaluate the benefits, risks, and disadvantages of beginning kidney replacement therapy. Particular clinical considerations and certain characteristic complications of kidney failure may prompt initiation of therapy before stage 5.
**Canadian Society of Nephrology (1999)**		
	< 12 mL/min/1.73 m^2^	When the GFR falls less than 12 mL/min/1.73 m^2^, look for symptoms or signs of uremia or evidence of malnutrition. If there is evidence of uremia, dialysis is recommended.
	< 6 mL/min/1.73 m^2^	When the GFR falls less 6 mL/min/1.73 m^2^, recommend initiation of dialysis.
**Australia: CARI guidelines (2004)**		
	< 10 mL/min/1.73 m^2^	Commence dialysis when GFR falls below approximately 10 mL/min/1.73 m^2^ if there is evidence of uraemia or its complications such as malnutrition.
	< 6 mL/min/1.73 m^2^	If there is no evidence of uraemia or its complications commence dialysis when GFR falls below approximately less than 6 mL/min/1.73 m^2^
**European Best Practice (2005)**		
	< 15 mL/min/1.73 m^2^	Dialysis should be instituted whenever the GFR is less than 15 mL/min/1.73 m^2^ and there is one or more of the following: symptoms or signs of uraemia, inability to control hydration status or blood pressure, or a progressive deterioration in nutritional status.
	< 6 mL/min/1.73 m^2^	In any case, dialysis should be initiated before the GFR has fallen to 6 mL/min/1.73 m^2^, even if optimal pre-dialysis care has been provided and there are no symptoms.
**UK: Renal Association (2009)**		
	< 15 mL/min/1.73 m^2^	We recommend that the decision to start RRT in patients with CKD stage 5 should be based on a careful discussion with the patient of the risks and benefits of RRT taking into account the patient’s symptoms and signs of renal failure. We suggest that serious consideration should be given to innitiating renal replacement therapy in patients
	< 6 mL/min/1.73 m^2^	With an eGFR less than 6 mL/min/1.73 m^2^, even if the patient is asymptomatic.

To arrive at a definitive answer, at this point there are only prospective trials. The first randomized study on the early or late start of dialysis was conducted. The results of the IDEAL study (Initiating Dialysis Early and Late) were published in 2010 (26).

The trial provided for the randomization of 828 patients with GFR values included between 10-15 mL/min/1.73 m^2^, split into two groups coming from 32 centers in Australia and New Zealand. In the early start subgroup, 404 patients were randomized for whom dialysis was set to initiate, their GFR was between 10 to 14 mL/min/1.73 m^2^ while in the other subgroup, 424 patients for whom dialysis was set to start, the GFR was between 7 and 5 mL/min/1.73 m^2^.

The GFR was calculated with the Cockcroft-Gault formula, adjusted for body surface and compared the MDRD formula. The patients belonging to the late start group were clinically monitored and initiated the replacement treatment if the physician deemed it necessary, following to analyzing their clinical progress. The primary outcome of the study was the mortality for any reason, while secondary objectives were represented by cardiovascular and infective events, along with dialysis complications.

The survival analysis has not found any statistically significant differences between the two groups in a follow-up period of 3.59 years, and none of the secondary outcomes was influenced in any significant way by the early or delayed start to dialysis.

An analysis of the trial, which was only revealed following to 10 months, took into consideration other aspects, i.e., economic and the quality of life which were evaluated with the assessment of quality of life (AQoL) and the 36-item short form health survey (SF-36) (27).

The results revealed that the early start of dialysis was associated with a higher quality of life, but also to an increase of costs: for dialysis, transportation and a greater global cost when compared to the patients in the late start group. These results are consistent with the results from a study of a more limited scope which found a significant economical advantages in postponing dialysis by optimizing the conservative diet-pharmacological approach in the elderly (age > or = 70 years old) without negative consequences in terms of mortality and morbidity (28).

These findings, coming from a recognized randomized trial such as the IDEAL study, contravene the convictions held so far in the nephrology, subverting what in the last two decades had been universally recommended by the principal international guidelines, which is that an early start of dialysis is needed to ward off the appearance of signs and symptoms of an advanced uremia.

However, if we analyze the results of the IDEAL study in a more detailed manner, the difference between the GFR when starting dialysis between the 2 groups is more limited as compared to the targets foreseen by the protocol. If as much as 76% of the randomized patients in the late start group had to wait for the dialysis start based on the appearance of uremic symptoms, these "protocol violations", even being legitimized by the study's design, constructed it so that the average GFR at the beginning of dialysis in the late group was of 9.8 mL/min, well over the late start target according to the protocol (5-7 mL/min).

In conclusion, the real difference of dialysis entrance GFR between the two groups has been an average of 2.2 mL/min, which is a modest difference and that could reshape the almost "provocative" intent of the trial. This difference made it possible in patients to live an average of 6 months free from dialysis without negative consequences while beginning dialysis on the basis of a predefined value of GFR has not improved the outcomes. According to the IDEAL study, delaying the beginning of dialysis to the appearance of uremic symptoms is a safe approach only if there is a good clinical-laboratory surveillance and if particular attention is dedicated to the a more adequate timetable for the creation of the vascular access or the implantation of the peritoneal catheter.

The lack of evidence of a better clinical "outcome” may cause the nephrologist to delay the innitiation of dialysis; the fewer benefits than expected as opposed to complications related to treatment, plus the increased costs and lack of beds often contribute to this circumstance on the part of the physician. In addition, patients are increasingly older and have underlying conditions that lead to cardiovascular instability intradialytic and difficulties creating and preserving vascular access.

A recent provocative trial may assist physicians postponing the innitiation of dialysis treatment in elderly patients (29). One hundred and twelve patients over 70 years old and with a GFR between 5-7 mL/min without uremic symptoms, were randomized to start dialysis treatment or a very low protein diet supplemented with keto-analogues to assess the non-inferiority of diet versus dialysis in one year mortality.

Forty patients in the diet group started dialysis treatment because of either fluid overload or hyperkalemia. There were 31 deaths (55%) in the dialysis group and 28 deaths (50%) in the diet group. The survival rates observed throughout a year which was 83.7% in the dialysis group versus 87.3% in the diet group.

Of the 56 patients in the diet group, 71% started dialysis after an average of 10 months; hospitalization and length of hospital stay were not different between groups. The diet also did not seem to affect nutrition per se. These results would seem to offer a temporary but effective alternative to dialysis with benefits for both the patients and the overloaded health system.

## 5. Conclusion

In the last years, in western countries, there has been a progressive change in the characteristics of patients that join the end-stage renal disease. These patients are usually older and affected by multiple comorbidities. Concurrently, the economic constraints induce the health systems to manage their resources even more carefully.

Knowing this, the nephrologist has the delicate task of deciding if, when and how to begin dialysis. The physician had assistant from the main guidelines which makes recommendations as to who and when treatment is proscribed. In the more recent literature, a careful evaluation of the patient who has reached the stage V of CKD-K/DOQI is required, and a judgement call since the outcome of the patient does not necessarily mean an early start of the dialysis treatment.

We believe the choice of when to initiate dialysis is tremendously important, as an adequate preparation of the patient himself, especially those in the early phases of CKD. The early referral to a nephrologist guarantees complete and correct information of the various aspects of the renal replacement treatment, and therapy can be started immediately to slow down the progression of the renal disease. The nephrologist will also investigate any potential uremic signs and symptoms, which enables the intervention of other specialists such as a psychologist, a dietitian or a social worker. Through this process, the best time for the creation of vascular or peritoneal access can be determined.
